# Supplementary material for: Prognostic Factors for Post-COVID-19 Syndrome: A Systematic Review and Meta-Analysis
Source: J Clin Med. 2022 Mar 11;11(6):1541. doi: 10.3390/jcm11061541 (PMC8948827; doi:10.3390/jcm11061541)
Supplement: Supplementary file 1 [file jcm-11-01541-s001.zip › Table S1_search strategy.pdf]

SEARCH STRATEGY FOR PUBMED

1. "COVID-19"[All Fields] OR "COVID-19"[MeSH Terms] OR "sars cov 2"[All Fields] OR "sars cov 2"[MeSH Terms] OR "Severe Acute Respiratory Syndrome Coronavirus 2"[All Fields]

2. "Long covid"[All Fields] OR "long-haulers"[All Fields]OR "long haulers"[All Fields] OR ongoing[All Fields] OR "ongoing symptoms"[All Fields] OR persistent[all fields] OR "post COVID 19 syndrome"[All Fields] OR "post-acute"[All Fields] OR "long-term effects"[All Fields] OR "long-term manifestations"[All Fields] OR consequence[All Fields] OR consequences[All Fields] OR "complications"[MeSH Subheading] OR complications[All Fields] OR sequelae[All Fields] OR sequela[All Fields] OR lingering[All Fields] OR "quality of life"[all fields]

3. "incidence"[MeSH Terms:noexp] OR "mortality"[MeSH Terms] OR "follow up studies"[MeSH Terms:noexp] OR "prognos*"[Text Word] OR "predict*"[Text Word] OR "course*"[Text Word] OR "risk factors"[all fields] OR "risk factor"[all fields]

4. Discharge[all fields] OR discharged[all fields] OR "post-discharge"[all fields] OR "post-hospitalization"[all fields] OR "post hospitalization"[all fields] OR "post-hospitalisation"[all fields] OR "post hospitalisation"[all fields]

5. 1 AND 2 AND 3 AND 4

NOTE ON STRATEGY DEVELOPMENT

The four components of the strategy were constructed as follows:

1. To retrieve publications on COVID-19, we adapted the string available on PubMed, to make it more specific to the objective of the review (eg. We excluded keywords on vaccines and diagnostic tests).

https://pubmed.ncbi.nlm.nih.gov/help/#clinical-study-category-filters

2. To identify studies on long-term effects of COVID-19, we selected keywords taken from literature reviews on the subject, particularly the work by Lopez Leon et al [1] and the NICE guidelines on long COVID [9].

3. To restrict results to prognostic studies, we used the appropriate string provided by PubMed

https://pubmed.ncbi.nlm.nih.gov/help/#clinical-study-category-filters

To this, we added the terms "risk factors", which were not included.

4. To restrict to the population of interest, patients discharged from hospital, we created the appropriate string.
