# Supplementary material for: Prognostic Factors for Post-COVID-19 Syndrome: A Systematic Review and Meta-Analysis
Source: J Clin Med. 2022 Mar 11;11(6):1541. doi: 10.3390/jcm11061541 (PMC8948827; doi:10.3390/jcm11061541)
Supplement: Supplementary file 1 [file jcm-11-01541-s001.zip › Table S2_QUIPS assessment.pdf]

Supplemental Table S2 –Risk of bias assessment for the six domains of the QUIPS tool for each study

First author	1. Study participation	2. Study attrition	3. Prognostic factor measurement	4. Outcome measurement	5. Adjustment for other prognostic factors $	6. Statistical analysis and reporting
Bellan et al [17]	Moderate	High	Low	Low	Low	Low
Blanco et al [18]	High	High	Moderate	Moderate	Low	Moderate
Eloy et al [19]	Low	High	Low	Low	Low	Low
Fernández-de-Las-Peñas et al [20]	Moderate	Low	Low	Low	Low	Low
García-Abellán et al [21]	Low	High	Moderate	Moderate	Low	Low
Ghosn et al [22]	Low	Moderate	Moderate	Moderate	Low	Moderate
Huang et al [23]	Moderate	Moderate	Low	Low	Moderate	Low
Li et al [24]	High	High	Low	Low	Moderate	High
Liang et al [25]	Moderate	High	Low	Low	Moderate	Moderate
Meije et al [26]	Moderate	Moderate	Low	Low	Low	Moderate
Munblit et al [27]	Moderate	Moderate	Low	Low	Low	Low
Romero-Duarte et al [28]	Low	Low	Low	Low	Moderate	Moderate
Shang et al [29]	Low	Moderate	Low	Low	Moderate	Moderate
Tarsitani et al [30]	Moderate	Moderate	Moderate	Moderate	Low	Low
Todt et al [31]	Low	High	Low	Low	Moderate	Moderate
van den Borst et al [32]	High	Moderate	Low	Low	Moderate	High
Wu et al [33]	Moderate	Moderate	Low	Low	Low	Moderate
Xiong et al [34]	Moderate	High	Moderate	Moderate	High	High
Zhang et al [35]	Low	High	Low	Low	Moderate	Low
Zhao et al [36]	High	High	Low	Low	Moderate	Moderate

$ **Explanations**: Risk of bias for this domain was assessed considering four elements: absence of multivariable analysis (High); confounding assessment not applied to outcomes and/or PFs of interest (Moderate); adjustment performed without specifying confounding (Low); multivariable analysis performed with variables of interest for the meta-analysis (not applicable).
