# Supplementary material for: Prognostic Factors for Post-COVID-19 Syndrome: A Systematic Review and Meta-Analysis
Source: J Clin Med. 2022 Mar 11;11(6):1541. doi: 10.3390/jcm11061541 (PMC8948827; doi:10.3390/jcm11061541)
Supplement: Supplementary file 1 [file jcm-11-01541-s001.zip › Table S3_acute disease severity.pdf]

SUPPLEMENTAL TABLE S3: Definitions of Acute Disease Severity reported in the publications of the studies investigating this prognostic factor

**First author**	**Outcome for which asociation with Acute Disease Severity was investigated**	**Definition of acute disease severity**
Bellan et al [17]	Respiratory	ICU admission
Blanco et al [18]	Respiratory	Patients were divided into mild (mild and moderate) and severe groups according to the severity of their symptoms during their hospital stays. The mild group did not have pneumonia imaging, the moderate group showed pneumonia, and the severe group had dyspnoea, respiratory frequency ≥30/minute, blood oxygen saturation ≤93%, arterial oxygen partial pressure/fractional inspired oxygen ratio (PaO2/FiO2) <300, and/or lung infiltrates >50% of the lung field within 24–48 hours
Li et al [24]	Respiratory	The severity of COVID-19 was graded according to the China National Health Commission Guidelines for Diagnosis and Treatment of SARS-CoV-2 infection. Laboratory confirmed patients with fever, respiratory manifestations and radiological findings indicative of pneumonia were considered as the moderate cases. Laboratory confirmed patients with any of the following conditions were considered to have severe COVID-19: (i) respiratory distress (respiration rate ≥ 30/min; (ii) resting oxygen saturation ≤ 93%, and (iii) arterial oxygen partial pressure (PaO2) / fraction of inspired oxygen (FiO2) ≤ 300 mmHg (1 mmHg = 0.133 kPa). Laboratory confirmed patients with any of the following conditions, such as (i) respiratory failure requiring mechanical ventilation, (ii) shock, and (iii) failure of other organs requiring intensive care unit (ICU), were considered to be in critical condition.
Liang et al [25]	Respiratory	"Disease severity status", definition not reported
Meije et al [26]	Respiratory	The patients were divided into three groups according to their worst ratio of partial pressure of oxygen in arterial blood to fraction of inspired oxygen (Pao 2/Fio 2) during hospitalization: Pao 2/Fio 2 >300, Pao 2/Fio 2 300–200 and Pao 2/Fio 2 <200.
Shang et al [29]	Respiratory; Fatigue	The severity of COVID-19 was defined according to the Chinese management guideline for COVID-19 (version 7.0). The definition of severity was as follows: mild (i.e. mild clinical symptoms without imaging feature of pneumonia); moderate (i.e. clinical symptoms such as fever and cough, with imaging feature of pneumonia); severe (i.e. dyspnoea, respiratory frequency ≥ 30/min, blood oxygen saturation ≤ 93%, partial pressure of arterial oxygen to fraction of inspired oxygen ratio < 300 and/or lung infiltrates > 50% within 24 to 48 hours); and critical ill cases (i.e. respiratory failure, septic shock and/or multiple organ dysfunction or failure). In this analysis, severe cases included the severe and critical ill classification in the 7th edition.
van den Borst et al [32]	Respiratory	World Health Organization criteria were applied to divide patients into mild, moderate, severe, or critical disease categories.
Xiong et al [34]	Respiratory; Fatigue	"Disease severity status", definition not reported
Zhang et al [35]	Fatigue	The disease severity was defined by World Health Organization guideline for COVID 19. Severe pneumonia refers to fever or suspected respiratory infection, plus 1 of the following: respiratory rate greater than 30 breaths per minute; severe respiratory distress; or oxygen saturation as measured by pulse oximetry (SpO2) less than or equal to 93% on room air.
Zhao et al [36]	Respiratory; Fatigue	Pneumonia
